# Retraction: Crosstalk between ATF4 and MTA1/HDAC1 promotes osteosarcoma progression

**DOI:** 10.18632/oncotarget.28887

**Published:** 2026-05-20

**Authors:** Heng Zeng, Jin-ming Zhang, Yu Du, Jiang Wang, Ye Ren, Mi Li, Hao Li, Zhuo Cai, Qian Chu, Caihong Yang

**Affiliations:** ^1^Department of Orthopedics, Tongji Hospital, Huazhong University of Science and Technology, Wuhan, Hubei 430030, P.R. China; ^2^Department of Orthopedic Surgery, The Second Affiliated Hospital, Chongqing Medical University, Chongqing 400016, P.R. China; ^3^Department of Oncology, Tongji Hospital of Huazhong University of Science and Technology, Wuhan, Hubei 430030, P.R. China; ^*^These authors have contributed equally to this work

**This article has been retracted:** Oncotarget has concluded its investigation of this paper, that identified several significant issues regarding data integrity:

- Figure 1G: The representative mouse lung control image and H&E staining appear to be duplicated from Figure 5D of an earlier, unrelated paper [1]. Additionally, the ATF4 mouse lung image is a duplication of the Figure 3D image from reference [1].
- Figure 1B: The IP/western blot for ATF4 and Actin were identified in a later publication [2] as HnRNPK and Tubulin in Figure 5E.
- Figure 2B: The western blot for Flag-ATF4 was found as the c-Myc blot in Figure 5A of reference [3].
- Figure 2C: The western blot for 143B/ATF4 is a duplicate of the MTA1 blot. Furthermore, the ZOS cells IP:ATF4/WB:MTA1 is a duplicate of IP:MTA1/WB:ATF4.
- Figure 4B: The western blot for ATF4 in the input was identified as the RioK1 blot in Figure 7H of a later publication [4].
- Figure 5A, 5C: The IP/WB for the β-TrCP blot was duplicated as the GST-ATF4-ODD blot in Figure 5C.
- Figure 6A: The western blot for b-actin was found in Figure 8C of reference [5] as the SW480/tubulin blot.

The authors did not respond to requests for comment or provide the original data. Consequently, the editorial decision has been made to retract this paper. Oncotarget has reached out to all authors to notify them of this retraction but has received no response.

Original article: Oncotarget. 2016; 7:7329–7342. 7329-7342. https://doi.org/10.18632/oncotarget.6940
